# Reliability Analysis of Traditional and Ballistic Bench Press Exercises at Different Loads

**DOI:** 10.1515/hukin-2015-0061

**Published:** 2015-10-14

**Authors:** Amador García-Ramos, Paulino Padial, Miguel García-Ramos, Javier Conde-Pipó, Javier Argüelles-Cienfuegos, Igor Štirn, Belén Feriche

**Affiliations:** 1 Faculty of Sport Sciences, University of Granada.; 2 Faculty of Sport, University of Ljubljana.; 3 Spanish Sport Council, Sierra Nevada Training Centre.

**Keywords:** load-velocity relationship, standard error of measurement, coefficient of variation, intraclass correlation

## Abstract

The purpose of this study was to determine test–retest reliability for peak barbell velocity (Vpeak) during the bench press (BP) and bench press throw (BPT) exercises for loads corresponding to 20–70% of one-repetition maximum (1RM). Thirty physically active collegiate men conducted four evaluations after a preliminary BP 1RM determination (1RM·bw-1 = 1.02 ± 0.16 kg·kg-1). In counterbalanced order, participants performed two sessions of the BP in one week and two sessions of the BPT in another week. Recovery time between sessions within the same week was 48 hours and recovery time between sessions of different weeks was 120 hours. On each day of evaluation the individual load-velocity relationship at each tenth percentile (20–70% of 1RM) in a Smith machine for the BP or BPT was determined. Participants performed three attempts per load, but only the best repetition (highest Vpeak), registered by a linear position transducer, was analysed. The BPT resulted in a significantly lower coefficient of variation (CV) for the whole load–velocity relationship, compared to the BP (2.48% vs. 3.22%; p = 0.040). Test–retest intraclass correlation coefficients (ICCs) ranged from r = 0.94–0.85 for the BPT and r = 0.91–0.71 for the BP (p < 0.001). The reduction in the biological within-subject variation in BPT exercise could be promoted by the braking phase that obligatorily occurs during a BP executed with light or moderate loads. Therefore, we recommend the BPT exercise for a most accurate assessment of upper-body velocity.

## Introduction

Ballistic exercises (e.g. a bench press throw [BPT] or a jump squat) are preferred during power training as athletes are able to generate higher values of velocity, power, force, and muscle activation in comparison to similar traditional resistance training exercises (e.g. a bench press [BP] or a squat) (Newton et al., 1996). The obligatory deceleration phase that occurs during a traditional resistance training exercise seems to be responsible for these results as described before (Cormie et al., 2011). It has recently been reported that when light and medium loads are lifted during a BP, the deceleration at the end phase of the movement is greater than what would be expected, due solely to the effect of gravity (Sánchez-Medina et al., 2010). This means that athletes must activate their antagonist muscles in order to apply force in the opposite direction to the load motion in order to stop the movement (Jarić et al., 1995). This persuades many coaches and researchers to recommend the inclusion of ballistic exercises rather than traditional resistance training exercises in power training programmes taking into consideration that ballistic exercises are generally more sport-specific and, therefore, may prompt adaptations that allow for greater transfer to performance (Cormie et al., 2011; McBride et al., 2002; McEvoy and Newton, 1998; Newton et al., 1999).

Despite the limitations previously discussed, it is common that the BP is the selected exercise to assess and monitor upper-body power (Marques et al., 2007; Morouço et al., 2011). According to Abernethy et al. (1995) the main goals of assessing strength and power are: (a) to identify specific deficiencies; (b) to identify individuals who may be suited to a particular athletic endeavour (talent identification); (c) to estimate the relative significance of strength and power to particular athletic pursuits; and (d) to monitor the effects of training interventions. In any case, it seems a necessary requirement that the chosen exercise must have both good concurrent validity and retest reliability (Hopkins, 2000). Validity concerns the agreement between the observed value and the true or criterion value of a measure (Hopkins, 2000). Therefore, if our intention is to measure the maximum capacity of an athlete, it does not seem reasonable to choose a traditional exercise as this does not allow the athlete to develop their fullest potential (Newton et al., 1996).

On the other hand, retest reliability concerns the repeatability of the observed value when the measurement is repeated (Hopkins, 2000). A reliable measurement needs both relative and absolute consistencies. Relative consistency implies the stability in the position of an individual within a group and is usually assessed using the intraclass correlation coefficient (ICC) (Weir, 2005). Therefore, relative consistency is more appropriate for talent identification. However, what is more important for discriminating the effects of a training program is that the measure has a good absolute consistency (measured as standard error of measurement [SEM] or coefficient of variation [CV]), which is related to the consistency of individual scores (Hopkins, 2000). Thus, it is important that athletes perform the assessed exercises with proper technique in order to be confident that a change in performance is truly an effect of the training period and is not due to motor learning in the exercise evaluated.

To our knowledge, there are no previous studies that compare test-retest reliability between the traditional resistance training exercise (BP) and the ballistic exercise (BPT) which are frequently used to monitor and assess upper-body power. Therefore, the purpose of this study was to determine test–retest reliability for peak barbell velocity during the BP and BPT exercises for loads corresponding to 20–70% of one-repetition maximum (1RM). We hypothesized that BPT might be a more reliable exercise promoted by the unnatural braking phase that obligatorily occurs during a traditional BP. These findings should provide valuable information to coaches and practitioners regarding the most accurate exercise for assessing the effect of a training intervention on the development of maximum velocity with a range of loads commonly employed during power training.

## Material and Methods

### Participants

Thirty physically active collegiate men (age: 21.7 ± 2.9 years; body height: 176.4 ± 5.3 cm; body mass: 74.7 ± 7.6 kg; body mass index: 24.0 ± 2.2 kg·m^−2^; 1RM BP: 75.7 ± 13.9 kg) volunteered to participate in this study. Selection criteria included (a) having at least six months of experience in BP training; (b) having no previous experience in BPT training; and (c) not consuming drugs, medications, or dietary supplements which influence physical performance. The study, which was conducted according to the Declaration of Helsinki, was approved by the University of Granada Institutional Review Board and, after being informed of the purpose and experimental procedures, participants meeting all criteria signed a written informed consent form.

### Measures

This study used a repeated measures design to compare test–retest reliability for peak barbell velocity (V_peak_) during the BP and BPT exercises performed on a Smith machine. After a preliminary testing session (body composition and maximal dynamic strength [1RM] in the BP), participants visited the laboratory on four separate occasions (twice a week, after 48 hours rest). They were randomly assigned with half of them performing both sessions of the BP in the first week and two sessions of the BPT in the second week, and vice versa. Individual load-velocity relationships at each tenth percentile (20–70% of 1RM) were determined on each day of evaluation. A linear position transducer (Real Power Pro Globus, Codogne, Italy) was used to measure barbell velocity with a frequency of 1,000 Hz. Relative (ICC) and absolute consistency (CV) were used to assess the repeatability of V_peak_ for each percentage of the 1RM in both exercises. Furthermore, paired samples t-tests and effect size (ES) were employed to establish differences in V_peak_ between the BP and BPT.

### Procedures

#### Preliminary testing (session 1)

Participants arrived at the laboratory after refraining from strenuous exercise for a minimum of 48 hours. As soon as they arrived, their body height (Seca 202, Seca Ltd., Hamburg, Germany) and mass (Tanita BC 418 segmental, Tokyo, Japan) were assessed. Before the commencement of the 1RM determination, participants selected the grip width that was the most comfortable, which was measured and kept constant throughout all testing sessions (García-Ramos et al., 2015). The warm-up consisted of joint mobility exercises, dynamic stretching and two sets of five repetitions with 20 kg and 30 kg, respectively. Participants then completed a progressive loading test for the determination of the 1RM in the BP. The initial load was set at 40 kg for all participants, and was progressively increased in steps of 10 to 1 kg, so that 1RM could be determined with a high level of precision. The heaviest load on the bar that each participant could properly lift was considered to be his 1RM. Participants performed 1–2 repetitions per load and the recovery time between attempts was at least five minutes. Participants needed an average of 5.2 ± 1.3 loading increments to achieve their 1RM. The 1RM presented high test-retest reliability (ICC = 0.95 and CV = 4.4%). The technique of execution was similar across the five days of evaluation. Participants started with their elbows fully extended and with their self-selected grip of the bar. Then, the barbell was lowered in a continuous motion of 1.5 s until the bar position was 1–2 cm above their intermammary line, and they were required to maintain this position for 1 s (velocity = 0 m·s^−1^). From that position, every participant was instructed to perform a purely concentric action (as quickly as possible) to regain the initial position. The duration of eccentric and isometric phases was administered by auditory feedback through an ad hoc audio file, while the concentric phase was always performed explosively, at the maximum possible speed. The only difference in the execution between the BP and BPT was that during the BPT participants accelerated the bar during the entire range of movement with the intention of throwing it as high as possible, whereas in the BP the bar had to be voluntarily decelerated at the end of the range in order to not throw it.

#### Reliability testing (sessions 2–5)

Participants visited the laboratory on four separate occasions during two consecutive weeks. In one week participants performed the two BP sessions, and in the other week they performed two BPT sessions. To compensate for a possible training effect, participants were counterbalanced. Recovery time between sessions within the same week was 48 hours and recovery time between sessions of the other week was 120 hours. Every session for the same participant was carried out at the same time of the day (to control circadian variation in performance) and under similar environmental conditions (22–23°c and 60% humidity). All tests were performed on a Smith machine (Technogym, Barcelona, Spain) in which the barbell was attached to both ends, with linear bearings on two vertical bars allowing only vertical movements. According to Vingren et al. (2011), a Smith machine without a counterbalance weight system was used in the present study.

Each testing session was preceded by a 10 min standardized warm-up, which included dynamic stretching, arm and shoulder mobilization and one set of six repetitions performed in an explosive manner with an external load of 17 kg in the exercise evaluated (BP or BPT). After warming up, participants rested for 5 min before starting the incremental loading test. Then, individual load-velocity relationships at each tenth percentile (20–70% of 1RM) were determined. Participants performed three repetitions (as quickly as possible) at each load, but only the best repetition, according to the criteria of the highest V_peak_, was considered for subsequent analysis. Recovery time between sets was at least 5 min. Two trained spotters were present on each side of the bar during the BPT and BP protocols to ensure safety, and to strongly encourage the participants throughout the test. V_peak_, which was defined as the maximum instantaneous value achieved during the concentric phase at a given load, was evaluated using a linear position transducer (Real Power Pro Globus, Codogne, Italy) at a sampling rate of 1,000 Hz. This device was interfaced to a personal computer and custom software (Globus Ergo System, V. 9.0.1) was used to automatically calculate the variable of interest (V_peak_).

### Statistical analysis

Data are presented as mean ± standard deviations (SD). Data normality was assessed using the Shapiro-Wilk test. A two-way (trial x load) repeated measures ANOVA was used to examine peak barbell velocity data for BP and BPT exercises. When appropriate, Bonferroni post hoc comparisons were performed. Relative reliability analysis was examined by the ICC_2,1_ (two-way random effect model). To examine absolute reliability, pairwise comparisons were first applied with the paired t-test to assess any significant differences in V_peak_ between trials 1 and 2 for each of the intensities (20–70% of 1 RM) analysed. The magnitude of between-session differences was also expressed as a standardized mean difference (Cohen’s d effect size; ES). The criteria to interpret the magnitude of the ES were as follows: <0.2 = trivial, 0.2–0.6 = small, 0.6–1.2 = moderate, 1.2–2.0 = large, and >2 = very large (Hopkins et al., 2009). Additionally, the SEM, calculated as the square root of the mean square error term from the ANOVA, was expressed both in absolute terms (m·s^−1^) and as a percentage of the participants’ mean scores (CV) to assess absolute consistency. The Pearson product moment correlation coefficient (*r*) was used to determine the relationship of V_peak_ between the BP and BPT for the same load. Finally, paired samples t-tests and ES were employed to establish differences in V_peak_ between the BP and BPT. Significance was accepted at *p* ≤ 0.05. All statistical analysis was performed using SPSS version 20.0 (SPSS, Chicago, IL).

## Results

The results from the two-way repeated measures ANOVA indicated there was no significant trial x % 1RM interaction (*p* = 0.768 for the BP and *p* = 0.490 for the BPT), no main effect for trial (*p* = 0.142 for the BP and *p* = 0.991 for the BPT), but there was a significant main effect for % 1RM (*p* < 0.001 for the BP and BPT). Bonferroni comparison showed that V_peak_ was higher at 20% of 1RM for both the BP and BPT, and significantly decreased with each subsequent intensity examined (i.e., 20% > 30–70%; 30% > 40–70%; 40% > 50–70%; 50% > 60–70%; 60% > 70%).

**Figure 1:**
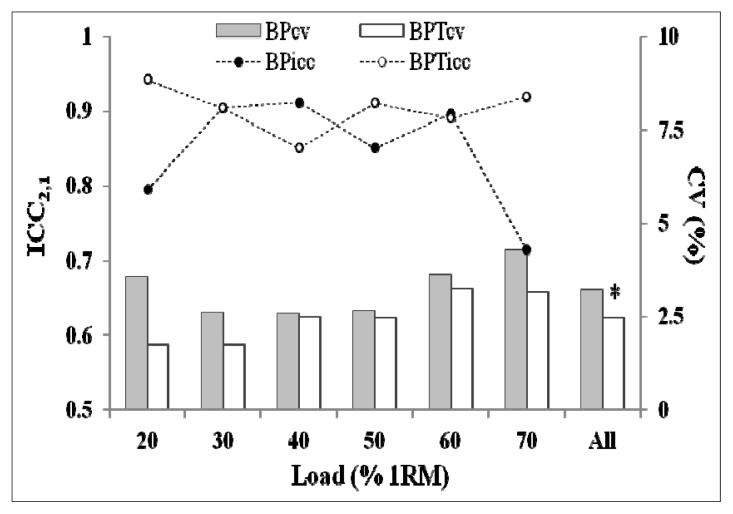
Reproducibility of peak barbell velocity measures at different percentages of one-repetition maximum (20–70% of 1RM) and for the load–velocity relationship as a whole (All). ICC, Intraclass correlation coefficient; CV, Coefficient of variation; BP, Bench press; BPT, Bench press throw; % 1RM, Percentage of one-repetition maximum; *, Significantly lower (p < 0.05) than the BP.

The results from each of the paired samples t-tests (Table 1) indicated that there were no mean differences in V_peak_ between trials 1 and 2 for any of the exercises (BP or BPT) and intensities (20–70% of 1RM) analysed. Furthermore, the ES of both exercises were trivial (<0.2) in all loads. On the other hand, despite the fact that the results of relative (ICCs = 0.94–0.85 and 0.91–0.71 for the BPT and BP, respectively) and absolute consistency (CV = 1.8–3.2% and 2.6–4.3% for the BPT and BP, respectively) showed good reliability of both exercises, the CV for the whole load–velocity relationship was significantly lower for the BPT compared to the BP (2.48% vs. 3.22%, respectively; *p* = 0.040) (Figure 1).

**Figure 2:**
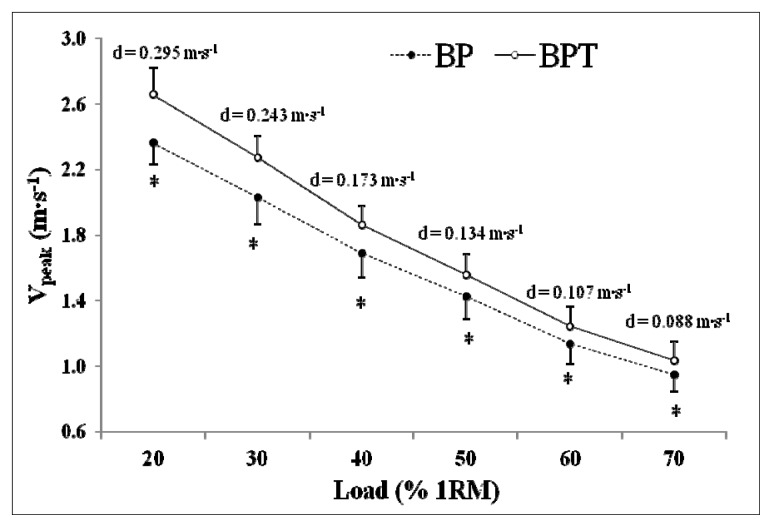
Mean ± SD peak barbell velocity values for the bench press and bench press throw exercises at each of the intensities examined. BP, Bench press; BPT, Bench press throw; V_peak_, Peak velocity; % 1RM, Percentage of one-repetition maximum; d, Absolute differences between BPT and BP. *, Significantly lower (p ≤ 0.001) than the BPT.

Considering mean scores from trials 1 and 2, significantly greater values of V_peak_ were obtained in the BPT for all loads employed (*p* < 0.001). However, differences between both exercises tended to decrease as the load increased (Figure 2). The ES was large for 20% (1.84), 30% (1.87) and 40% (1.51) of 1RM and moderate for 50% (1.08) 60% (0.90) and 70% (0.76) of 1RM.

## Discussion

The aim of the present study was to compare test-retest reliability of peak barbell velocity in the two exercises most frequently used to assess upper-body power. Our results indicate that both exercises have high reliability in terms of relative (ICC) and absolute consistency (CV). As indicated by Weir (2005), ICC concerns the ability of a test to differentiate between different individuals. Nevertheless, the SEM or “typical error”, as named by Hopkins (2000), may have more usefulness to practitioners, such as strength coaches, as it quantifies the precision of individual scores on a test, providing an absolute index of reliability. For comparative purposes, in sports science SEM is usually expressed as CV (percentage of subjects mean scores) (Hopkins, 2000). In this context, the main findings of this investigation was the lower CV obtained for the BPT exercise for both the whole load–velocity relationship (BPT = 2.48% and BP = 3.22%; *p* = 0.040) and for each specific load (Table 1), suggesting that probably the BPT should be the preferred exercise when coaches want to accurately measure the effects of a training period.

The first step in a reliability study is to rule out the presence of systematic bias (Atkinson and Nevill, 1998). Systematic bias involves a tendency of the retest measurement to be higher (e.g. learning effect) or lower (e.g. fatigue) regarding a previous test. Our results from each of the paired samples t-tests (Table 1) indicated that there were no mean differences in V_peak_ between trials 1 and 2 for any of the exercises (BP or BPT) and intensities (20–70% of 1RM) analysed. However, the absence of significant differences does not necessarily imply good reliability, precisely because a large amount of random error between tests may promote the absence of these differences (Bland and Altman, 1986). Nevertheless, the ES between trials 1 and 2 were always trivial (<0.2) in both exercises at all intensities, reinforcing the absence of systematic bias. Therefore, it seems that 48 hours was enough time to induce optimal individual recovery and that a learning effect in performing the BPT did not appear, despite the fact that participants had no previous experience performing this exercise.

A goal of power training should be that athletes can apply more force with the same absolute load, or in other words, develop higher velocities at the same absolute load. In this context, the determination of the individual load-velocity (or load-power) relationship is one of the tests most commonly used to assess the effects of a training intervention (Cormie et al., 2010; Vuk et al., 2012). Surprisingly, only a few studies have examined the reliability of these measurements in the BP exercise. Stock et al. (2011) calculated test–retest reliability in the free-weight BP exercise for V_peak_ at loads corresponding to 10–90% of 1RM. Compared with this study, our results revealed higher reliability in terms of relative (ICC) and absolute (CV) consistencies. We think that these results could be promoted mainly for four reasons: (a) the type of exercise: free weight vs. Smith machine. The Smith machine employed in our study increases reliability by allowing only vertical movements; (b) the number of repetitions performed per load: single repetitions vs. the best of three repetitions. The greater number of repetitions performed in our study increases the chances of obtaining the maximum performance from the individual; (c) the pattern of movement: eccentric-concentric vs. eccentric-isometric-concentric. To impose a momentary pause between the eccentric and concentric actions allows more reproducible, consistent measurements (Pallarés et al., 2014); (d) the motion sequence: free vs. audio file. The use of an ad hoc audio file may increase measurement reproducibility by standardizing the durations of the eccentric and isometric phases.

On the other hand, this is the first study that compares test-retest reliability between the traditional resistance training exercise (BP) and the ballistic exercise (BPT) most frequently used to monitor and assess upper-body power. A good measurement is required to have both concurrent validity and retest reliability (Hopkins, 2000). On the one hand, it seems contradictory that upper-body power is evaluated with BP exercise as athletes cannot develop their fullest potential (Cormie et al., 2011). In addition, our results showed that the BPT was also the most reliable exercise. The unnatural deceleration phase that obligatorily occurs during all traditional resistance training exercises (Cormie et al., 2011) may be responsible for the lower reliability of the BP. This would explain that the greatest differences between exercises occur with light loads (20–30% of 1RM), as these are the loads with larger braking phases (Sánchez-Medina et al., 2010). On the contrary, the strength association of V_peak_ between both exercises (BP and BPT) has a tendency to increase as the load is increasing. Therefore, it seems that the BPT is more reliable even in subjects without previous experience in this exercise.

It has been known for some time that the BPT allows for the development of higher values of velocity, power, force, and muscle activation in comparison to BP exercise (Newton et al., 1996). Our results concur, showing significantly greater values of V_peak_ in loads ranging from 20 to 70% of 1RM. As expected, differences between both exercises tend to decrease as the load is increased (Figure 2) confirming that the benefits of ballistic exercises are magnified with the lowest loads. Therefore, we also share the view of other authors that recommend training with ballistic exercises (e.g. BPT) rather than traditional resistance training exercises (e.g. BP) for the development of muscular power, as ballistic exercises may prompt adaptations that allow for greater transfer to sport performance, due to the greater specificity of the movement and because they represent a higher mechanical stimulus (Cormie et al., 2011; Newton et al., 1996).

In order to mitigate the effects of potentiation or fatigue the sequence of loads during loaded tests tend to be randomized (Cuk et al., 2014; Sreckovic et al., 2015). Notwithstanding, our assessment protocol included progressive loads, following previous studies that had already assessed test-retest reliability during the BP exercise (Pallarés et al., 2014; Stock et al., 2011). While the sequence of loads could compromise the comparison among different loads (e.g., 20%RM vs 30%RM), we believe that the non-randomized order of the loads should not be a confounding factor for the reliability analysis conducted in the present study, since all participants followed the same order during the four days of testing. One of the strengths of the present study was the counterbalanced design of the two exercises (BP and BPT). This design allowed us to compare V_peak_ values between exercises and was also a key factor to overcome the learning process.

In summary, due to the higher reliability scores found in the BPT, we recommend this exercise for the assessment and monitoring of upper-body velocity even in subjects without previous experience in this exercise. Although the BP also has good reliability, it does not seem appropriate to choose this exercise since athletes cannot develop their fullest potential. It should be noted that our results may not be representative of other exercises used to assess lower-body power. Consistent with other studies, our results seem to show lower reliability during low-velocity movements (Brown et al., 2005; Pallarés et al., 2014; Stock et al., 2011). Further studies would develop the analysis of test-retest reliability of different mechanical variables (e. g. peak power, average power, average velocity, etc.) frequently used by researchers for the assessment of muscle power.

## Conclusions

A good measurement requires both validity and retest reliability (Hopkins, 2000). As indicated by both Hopkins (2000) and Weir (2005), high validity does not necessarily imply high test-retest reliability, and vice versa. On the one hand, higher validity of the BPT was demonstrated by Newton et al. (1996). These authors showed that the obligatory deceleration phase that occurs during the BP did not allow athletes to develop their fullest potential. In addition, our results suggest that the BPT is also the exercise with higher test-retest reliability. The cause of this result is likely to be the unnatural braking phase that obligatorily occurs during the BP exercise. The lower relationship of V_peak_ between the BP and BPT at 20–30% of 1RM could support this claim. Based on these results, we recommend the BPT exercise for the accurate assessment of upper-body velocity, even in subjects without previous experience in this exercise.

## Figures and Tables

**Table 1:** Test-retest reliability of peak barbell velocity during the bench press (BP) and bench press throw (BPT) exercises.

% 1RM	Exercise	Score	p	ES	ICC_2,1_	SEM	CV	r*
20	BP	2.364 ± 0.13	0.890	0.025	0.795	0.084	3.56	0.726
	BPT	2.658 ± 0.16	0.617	0.040	0.943	0.047	1.76	

30	BP	2.031 ± 0.17	0.931	0.007	0.905	0.053	2.61	0.752
	BPT	2.274 ± 0.13	0.098	0.125	0.904	0.040	1.77	

40	BP	1.690 ± 0.15	0.098	0.123	0.912	0.044	2.58	0.797
	BPT	1.864 ± 0.11	0.915	0.010	0.851	0.047	2.51	

50	BP	1.426 ± 0.14	0.562	0.041	0.851	0.038	2.67	0.806
	BPT	1.561 ± 0.12	0.795	0.019	0.911	0.038	2.46	

60	BP	1.139 ± 0.12	0.518	0.057	0.897	0.041	3.62	0.766
	BPT	1.246 ± 0.12	0.377	0.076	0.891	0.040	3.24	

70	BP	0.948 ± 0.10	0.108	0.162	0.714	0.041	4.29	0.808
	BPT	1.036 ± 0.12	0.134	−0.104	0.920	0.032	3.15	

Score, Subjects’ mean score ± SD (m·s^−1^); p, p values from a paired samples t-test between trials 1 and 2; ES, Effect size; ICC_2,1_, Intra-class correlation coefficient of two-way random effect model; SEM, Standard error of the measurement; CV, Coefficient of variation; r, Pearson’s coefficient of correlation between the BP and BPT;

* , significant correlation (p < 0.001).
